# Epidemiology of Musculoskeletal Injuries in Adult Athletes: A Scoping Review

**DOI:** 10.3390/medicina57101118

**Published:** 2021-10-17

**Authors:** Francesca Gimigliano, Giuseppina Resmini, Antimo Moretti, Milena Aulicino, Fiorinda Gargiulo, Alessandra Gimigliano, Sara Liguori, Marco Paoletta, Giovanni Iolascon

**Affiliations:** 1 Department of Mental and Physical Health and Preventive Medicine, University of Campania Luigi Vanvitelli, 81100 Naples, Italy; francesca.gimigliano@unicampania.it; 2 Centre for the Study of Osteoporosis and Metabolic Bone Disease, Section of Orthopaedic and Traumatology, Treviglio-Caravaggio Hospital, 24047 Bergamo, Italy; giuseppina_resmini@ospedale.treviglio.bg.it; 3 Department of Medical and Surgical Specialties and Dentistry, University of Campania Luigi Vanvitelli, 81100 Naples, Italy; milena.aulicino@studenti.unicampania.it (M.A.); fiorinda.gargiulo@studenti.unicampania.it (F.G.); sara.liguori@unicampania.it (S.L.); marco.paoletta@unicampania.it (M.P.); giovanni.iolascon@unicampania.it (G.I.); 4 Medical Direction, ASST Fatebenefratelli-Sacco, 20157 Milan, Italy; alessandra.gimigliano@asst-fbf-sacco.it

**Keywords:** musculoskeletal injuries, sport, Olympic Games, athletes, incidence, sprain, strain

## Abstract

*Background and Objectives*: Sport-related musculoskeletal injuries (MSK-Is) are a common health issue in athletes that can lead to reduced performance. The aim of this scoping review was to synthetize available evidence on injury incidence rates (IIRs), types, and sites that affect the musculoskeletal (MSK) system of adult athletes. *Materials and Methods*: We performed a scoping review on the Pubmed database limiting our search to 33 Olympic sports. *Results*: We identified a total of 1022 papers, and of these 162 were examined in full for the purpose of this review. Archery was the sport with the highest risk of injuries to the upper extremities, marathons for the lower extremities, and triathlon and weightlifting for the body bust. In the majority of the sports examined, muscle/tendon strain and ligament sprain were the most common MSK-Is diagnoses, while athletics, karate, and football were the sports with the highest IIRs, depending on the methods used for their calculations. *Conclusions*: Our scoping review highlighted the general lack and dishomogeneity in the collection of data on MSK-Is in athletes.

## 1. Introduction

Musculoskeletal injury (MSK-I) is a general term that includes any trauma that causes damage to muscles, bones, tendons, joints, ligaments, and other soft tissues. MSK-Is represents one of the most common health conditions in athletes, with consequences not only in terms of the lowering of performance, or withdrawal from competitions, but also of economic costs [1].

The International Olympic Committee (IOC) in its manual on sports injuries defined MSK-Is as “new or recurring musculoskeletal complaints incurred during competition or training that require medical attention, regardless of the potential absence from competition or training” [2]. 

The origin of the term athlete is from the Greek “athlos” that means achievement and serves to indicate an extraordinary physical performance [3]. In 2016, C.G.S. Araujo and J. Scharhag identified four criteria that should be simultaneously fulfilled to define an athlete for health and research purposes: “(a) to be training in sports aiming to improve his/her performance or results; (b) to be actively participating in sport competitions; (c) to be formally registered in a local, regional, or national sport federation as a competitor; and (d) to have sport training and competition as his/her major activity or focus of interest, almost always devoting several hours in all or most of the days to these sport activities, exceeding the time allocated to other professional or leisure activities” [3]. Athletes competing in Olympic Games are called Olympic Athletes.

Olympic Games, or the Olympics, are a set of international sporting competitions taking place every four years, each time in a different country. They represent the most important sporting competition in the world, with more than 200 nations involved. The Olympics are divided into summer and winter games which alternate every two years. Due to the COVID-19 health emergency, the 2020 summer Olympics, were rescheduled and took place from 23 July to 8 August 2021 in Tokyo, Japan. The official program for Tokyo 2020, approved by the IOC on 9 June 2017, included 339 events in 33 different sports, for a total of 50 disciplines [4]. 

During the previous summer Olympic Games (Rio de Janeiro, 2016), the medical staff reported 1101 injuries over the 17-day period for an overall proportion of 8% of the athletes that incurred at least one injury, which was slightly lower than in prior Olympic Games [5]. The sport with the highest proportion of athletes injured was BMX cycling (38%), while the ones with the lowest were canoe slalom, rowing, shooting, archery, swimming, golf, and table tennis (0–3%) [5]. 

Injury incidence data on MSK-Is in adult athletes are limited. In the majority of cases injury data are reported all together and it is difficult to extrapolate information related only to the MSK system. Moreover, there are several ways to calculate injury incidence rates (IIRs), therefore it is not possible to generalize the results of available epidemiological studies [6]. Among the other methods, three of the most popular ones are:

- injuries/1000 h exposures (/1000 h) which represents the number of injuries per 1000 h of exposures;
- injuries/1000 athlete-exposures (/1000 AEs), which is based on the total number of athletes exposed during a competition or training irrespective of their actual time of exposure [7]. An athlete-exposure is defined as “one athlete’s participation in one practice or game in which there is a possibility of sustaining an athletic injury” [6];
- injuries/1000 player-hours, represents the actual time of player-exposure that is the probability of injury for 1 player over 1000 h of total exposure” [7].

The aim of this paper was to synthetize available evidence on the IIRs, types, and sites of MSK-Is in adult athletes of Olympic Games sports.

## 2. Materials and Methods

In performing this scoping review, the PRISMA-ScR model was followed [8]. 

Sports and disciplines in the Olympic Games can vary from one edition to another, therefore, for the purpose of this scoping review we decided to limit our search to the 33 Olympic sports that would have taken place in the Tokyo 2020 edition. 

We ran a search on the PubMed database on 25 June 2021, using the following search strategy: (“Epidemiology”[Mesh] OR “Incidence”[Mesh]) AND (“Musculoskeletal System/injuries”[Mesh] OR “Athletic Injuries”[Mesh]) AND (“Water Sports/epidemiology”[Mesh] OR “Water Sports/injuries”[Mesh] OR “Swimming/epidemiology”[Mesh] OR “Swimming/injuries”[Mesh] OR “Diving/epidemiology”[Mesh] OR “Diving/injuries”[Mesh] OR archer* OR “athletics injuries” OR “Track and Field/injuries”[Mesh] OR “Sports/epidemiology”[Mesh] OR “Sports/injuries”[Mesh] OR “Racquet Sports/injuries”[Mesh] OR “badminton injuries” OR “Baseball/injuries”[Mesh] OR “Basketball/injuries”[Mesh] OR “Boxing/epidemiology”[Mesh] OR “Boxing/injuries”[Mesh] OR “Bicycling/injuries”[Mesh] OR “equestrian injuries” OR “fencing injuries” OR “Hockey/injuries”[Mesh] OR “Football/injuries”[Mesh] OR “Golf/injuries”[Mesh] OR “Gymnastics/injuries”[Mesh] OR “handball injuries” OR “Martial Arts/injuries”[Mesh] OR “modern pentathlon” OR sailing OR “shooting injuries” OR “Skating/injuries”[Mesh] OR “sport climbing” OR “table tennis” OR “Tennis/injuries”[Mesh] OR “triathlon injuries” OR “Volleyball/injuries”[Mesh] OR “Weight Lifting/injuries”[Mesh] OR “Wrestling/injuries”[Mesh]).

We limited the search to studies published from 1 January 2001 to 31 May 2021. We included papers with: (1) a population of adult (older than 18 y.o.) professional, non- Paralympic, athletes playing any of the 33 sports selected; (2) reporting information about the IIRs of MSK-Is was in one of the following formats: injuries/1000 h, injuries/1000 AEs, or injuries/1000 player-hours; (3) any study design; and (4) written in English.

The study selection and data extraction were done by two authors independently (MA, and FiG), and in case of any controversies, a third author (FG) was consulted.

## 3. Results

**Figure 1:**
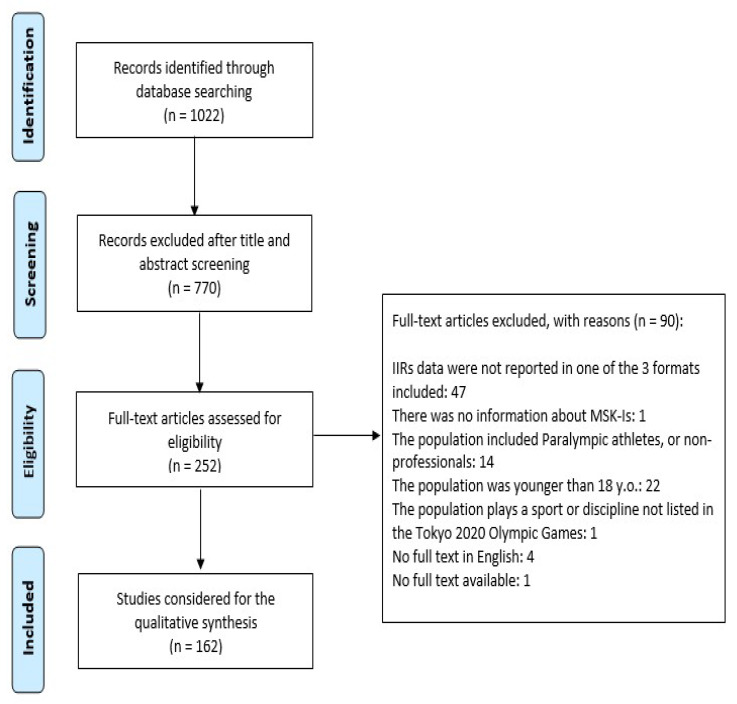
PRISMA-ScR flow chart of the study selection process.

We identified a total of 1022 papers with our search string (see the flow diagram, Figure 1).

Of these papers, 613 were about one of the 33 sports (50 disciplines) included and 67 dealt with more than one of these sports. As shown in Figure 1 the number of papers that met the inclusion criteria and were analysed for the purpose of this scoping review was 162. Table 1 reports the most recent and generalizable data on injuries per type of sport, including the most common MSK-Is sites (excluding the head and sometimes the neck if the data were given together with the head), types, and their IIRs. With the exception of marathons where specific MSK IIRs were available, in all other cases, data that are reported in Table 1 referred to any kind of injury. Of the 33 sports included, no data were available for badminton, canoeing, cycling, equestrian, modern pentathlon, rowing, shooting, skateboarding, surfing, and table tennis. When examining data related to injury sites, we can see that archery was the sport with the highest risk of injuries to the upper extremity, mainly to the shoulder of the dominant side. Marathon was the highest risk sport for the lower extremity, while triathlon and weightlifting were the highest risk for the body trunk. In the majority of the sports examined, muscle/tendon strains and ligament sprains were the most common MSK-Is. When considering the different IIRs reporting methods, for 12 disciplines data available on IIRs were expressed as injuries/1000 h exposures and archery (0.00536/1000 h) was the sport with the lowest IIR, while athletics (overall IIR for marathon—65.0/1000 h, 95% CI 61.4, 68.7) was the highest; the injuries/1000 AEs method was used in 13 disciplines and women’s gymnastics (1.40/1000 AEs, 95% CI 1.09, 1.71) had the lowest IIR vs. karate (88.3/1000 AEs, 95% CI 66.6, 117.2) which had the highest; in only four cases were IIRs reported as injuries/1000 player-hours and the lowest IIR was reported for handball (4.3/1000 player-hours) while the highest was for football (50.8/1000 player-hours, 95% CI 41.0, 60.6).

## 4. Discussion

To the best of our knowledge, this is the first review summarizing data that are related to the epidemiology, sites, and types of MSK-Is that are related to the sports included in the last Olympic Games. Sport is not only an activity that benefits participants’ physical and mental health, but it is first and foremost valuable in the context of social inclusion and integration.

Epidemiological data on MSK-I in athletes are limited. With the exception of marathons, all injury data reported in Table 1 are general and do not specifically refer to the MSK system. Moreover, the available data are difficult to generalize as there are several reporting methods for IIRs. Our study shows that depending on the reporting method the sports associated with the highest IIRs were athletics, karate, and football. We cannot say that these are the three sports with the absolute highest IIRs as we can see, for example, that for football this is only true when we refer to the data reported as injuries/1000 player-hours and not when they were expressed as injury/1000 h. The same is true for archery, gymnastics, and handball which were the sports with the lowest IIRs as measured with the three different methods, respectively. It would have been interesting to have more homogeneous data also in terms of the timing of the injuries (i.e., during training or competition), and the severity of the injuries, including if they resulted in time loss from sport or not.

Based on our findings, evidence suggests that archery and baseball were the sports with the highest risk of injuries to the upper extremity. Upper extremities seem to be more compromised in sports with a greater overhead involvement and in which the throwing mechanism can lead to overuse or acute microtraumas affecting, above all, the dominant shoulder and elbow [35]. On the contrary sports such as athletics (marathon), triathlon, football, gymnastics (women), basketball, pole vault, rugby sevens, volleyball (women), handball, and tennis have a risk of injuries affecting the lower extremities that is higher than 50%. These are all sports in which the lower limbs are more stressed. The knee was the most injured joint above all in competitive sports involving stop-start movements, direction changes, jumps, and landings, with or without passing and/or shooting a ball [36]. It has been shown that repetitive landing, dynamic knee abduction, and shallow knee flexion angles can cause medial collateral ligament, medial patellofemoral ligament, and anterior cruciate ligament injuries [37]. The body trunk had more homogeneous injury proportions across all examined sports. This might be due to the fact that it is not an anatomical site directly involved in any of these sports. As demonstrated in Table 1, the sports with the highest proportions of injuries affecting the body trunk were triathlon and weightlifting. 

In terms of the most common types of MSK-Is among professional athletes of Olympic sports and disciplines, muscle/tendon strains and ligament sprains were the most common MSK-Is. Sprains may be acute or chronic; an acute sprain is caused by a sudden injury that forces the joint beyond its functional range of motion, while a chronic sprain is due to repetitive movements that lead to an overuse injury [38]. Field hockey is one of the sports that is most commonly associated with sprains, especially at the ankle [39]. Balance training and foot dorsiflexor strengthening exercises and wearing ankle bracing might reduce the risk of ankle sprains [40,41]. Strain is the other most frequent injury in sports that require a major muscular effort. Up to 50% of injuries in field hockey are classified as strains. This is probably due to the fundamental requisite of dribbling the ball and moving rapidly in a semi-crouched posture [42]. As reported in Table 1 strains were very common injuries also in pole vault and swimming. In pole vault, muscular strain represents one of the most common causes of lower back injuries [13]. The injury mechanism might be related to the plant/takeoff movement that forces the spine into hyperextension as the athlete drives forward off the ground [13]. Swimmers, especially those who practice the breaststroke style, have an increased risk for strain injuries localized to the knee medial collateral ligament and hip flexors and adductor muscles [43]. Sport-related MSK-Is includes bone fractures which even though are less frequent they are considered as a major injury as they are associated with a greater rate of time loss from sport [44]. 

The main limitation of our scoping review is that we ran the literature search only on PubMed. Another important limit of the review, as well as of the existing literature on sport injuries, is that there are too many and not comparable ways to report IIRs. Moreover, even though MSK-Is represents one of the major health issues in professional athletes, there are only very limited data reporting selectively on MSK IIRs. 

The strength of the paper is that it gives an overview of sports injury risk rates and types. This could represent valuable information for athletes, coaches, and trainers that might adapt their training programs and competition strategies to reduce the risk of the most common and specific sport related injuries.

## 5. Conclusions

In recent years there is a greater awareness on the importance of the prevention of injuries in sport. If we compare the IIRs during football World Cups from the 1998 to the 2014 editions, we can see how the number of injuries has been constantly decreasing thanks to the improvement of preventive measures [20]. Sporting injuries should be systematically recorded in databases in a standardised way, thus allowing for data comparison. It is necessary to enforce interdisciplinary cooperation among all involved parties, including coaches, trainers, physiotherapists, physicians, clubs, and federations. Moreover, we believe that future literature should be focused not only on the structural components of the injuries but on the whole functioning profile of the athlete, and that the use of the bio-psycho-social framework proposed by the International Classification of Functioning, Disability, and Health might enrich our knowledge on this topic [45]. 

## Figures and Tables

**Table 1:** MSK-Is in Olympic Games.

Sport (Disciplines)	Injury Sites ^2^	Types of Injury	IIRs ^1,2^
Aquatic (Swimming [9], Marathon swimming, Diving, Artistic swimming, Water polo [10])	Swimming: upper extremity (38.7%), lower extremity (42.0%), body trunk (19.3%) Water polo: upper extremity (38.7%), lower extremity (18.8%), body trunk (16.9%)	Swimming: tendonitis (58%), strain (35.5%), and sprain (6.5%) Water polo: (sub)luxation/sprain (22.7%), strain (9.9%), tendinosis/arthritis/bursitis/impingement or similar (9.1%), fracture (5.1%), muscle spasm (4.0%), tendon/ligament rupture (1.1%)	Swimming: 3.04/1000 h (95% CI 2.04, 4.49) or 5.55/1000 AEs (95% CI 3.73, 8.18) Water polo: 56.2/1000 h (95% CI ± 6.74) competition
Archery [11]	Overuse: upper extremity (86.2%), body trunk (11.8%). Acute: upper extremity (83.4%).	Overuse: tendons, ligaments, and articulation injuries (67.9%) Acute: fractures (83.3%)	0.00536/1000 h (overuse and acute)
Athletics [12,13]	Marathon: lower extremity (92.6%) Pole vault: upper extremity (20.8%), lower extremity (59.8%), body trunk (18.1%)	Marathon: tendonitis (10.3%), strain (2.4%), sprain (2.3%), bursitis (1.0%) (major and minor injuries) Pole vault: strain (37.5%), sprain (18.1%), stress reaction (13.9%), tendinitis (11.1%), fracture (11.1%)	Marathon: 65.0/1000 h (95% CI 61.4, 68.7); major MSK-Is 0.8/1000 h (95% CI 0.4, 1.3), minor MSK-Is 11.2/1000 h (95% CI 9.8, 12.9) Pole vault: 7.9/1000 AEs (95% CI, 6.2, 10.0)
Badminton	Data not available	Data not available	Data not available
Baseball [14]/Softball [15]	Baseball: upper extremity (51.4%), lower extremity (30.6%), body trunk (11.7%) Softball: upper extremity (33.1%), lower extremity (42.1%), body trunk (9.8%)	Baseball: data not available Softball: ligament sprain, muscle-tendon strain (data not available)	Baseball: 3.61/1000 AEs (95% CI = 3.49, 3.74) Softball: 4.30/1000 AEs (95% CI = 4.13, 4.47) competition; 2.67/1000 AEs (95% CI = 2.57, 2.77) training
Basketball (3 × 3, basketball [16])	Men: upper extremity (18.5%), lower extremity (58.7%), body trunk (8.4%) Women: upper extremity (14.5%), lower extremity (63.1%), body trunk (6.4%)	Men: sprain (30.1%), strain (14.3%), tendonitis (3.8%), fracture (3.2%), dislocation (3.0%), spasm (2.6%), inflammation (1.8%) Women: sprain (29.0%), strain (16.7%), tendonitis (5.2%), inflammation (4.6%), dislocation (3.2%), fracture (3.0%), spasm (2.6%)	Men: 7.97/1000 AEs (95% CI 7.65, 8.30) Women: 6.54/1000 AEs (95% CI 6.22, 6.85)
Boxing [17]	Upper extremity (24.5%), lower extremity (15.6%), body trunk (14.1%)	Tear (12.0%), pain (7.8%), strain (4.2%), rupture (2.0%), fracture (1.0%)	12.8/1000 h (training)
Canoeing (slalom, sprint)	Data not available	Data not available	Data not available
Cycling (BMX freestyle, BMX racing, mountain bike, road, track)	Data not available	Data not available	Data not available
Equestrian (eventing, dressage, jumping)	Data not available	Data not available	Data not available
Fencing [18]	Upper extremity (42.9%), lower extremity (35.7%), body trunk (21.4)	Sprain (25.0%), Pain (25.0%)	2.43/1000 AEs
Hockey (field hockey [19])	Men: upper extremity (19.0%), lower extremity (41.0%), body trunk (4.0%) Women: upper extremity (14.0%), lower extremity (28.0%), body trunk (0.0%)	Data not available	36.2/1000 player-hours (95% CI 31.6, 40.8) Men: 48.3/1000 player-hours (95% CI 30.9, 65.8) Women: 29.1/1000 player-hours (95% CI 18.6,39.7)
Football (soccer [20])	Upper extremity (10%), lower extremity (65%), body trunk (7%)	Strain/muscle fibre rupture (24%), sprain/dislocation (8%), fracture (6%), tendon or ligament rupture/meniscus lesion (4%)	29.3/1000 h (95% CI 21.9, 36.7); 50.8/1000 player-hours (95% CI 41.0, 60.6)
Golf [21]	Upper extremity (40.7%), lower extremity (26.1%), body trunk (29.0%)	Tendinosis/tendinopathy (21.2%), ligament sprain (13.6%), meniscus lesions (11.2%), muscle strain, rupture, or tear (9.1%), and inflammation of unknown cause (7.0%)	8.5/1000 AEs (competition), and 3.3/1000 AEs (training)
Gymnastics [22] (artistic, rhythmic, trampoline)	Severe injuries: upper extremity (16.5%), lower extremity (64.5%), body trunk (8.9%)	Severe injuries: sprain (31.6%), strain (13.9%), fracture (13.9%), dislocation (8.9%), sub-luxation (3.8%), inflammation (2.5%)	Women: 1.40/1000 AEs (95% CI 1.09, 1.71)
Handball [23]	Upper extremity (28.7%), lower extremity (52.0%), body trunk (17.2%)	Sprain (26.5%), rupture (12.2%), strain (4.9%), fracture (4.4%), subluxation/dislocation (2.3%)	4.3/1000 player-hours
Judo [24]	Upper extremity (10.2%), lower extremity (9.7%), body trunk (10.9%)	Data not available	4.2/1000 h (training)
Karate [25] (kata, kumite)	Lower extremity (12.0%)	Data not available	88.3/1000 AEs (95% CI 66.6, 117.2)
Modern pentathlon	Data not available	Data not available	Data not available
Rowing	Data not available	Data not available	Data not available
Rugby (rugby sevens [26])	Upper extremity (21.4%), lower extremity (58.3%), body trunk (5.5%)	Joint sprains (25.2%), muscle injury (16.7%), tendon injury (12.1%), cartilage injury/impingement (9.3%), joint dislocation/instability (5.5%), bone injury (5.5%)	43.2/1000 player-hours (95% CI, 43.0–43.3)
Sailing [27]	Upper extremity (12%), lower extremity (23%), body trunk (29%)	Muscle cramp/spasm (20%), muscle strain (13%), sprain (13%), tendinopathy (13%)	0.59/1000 h
Shooting	Data not available	Data not available	Data not available
Skateboarding	Data not available	Data not available	Data not available
Sport climbing [28]	Lower extremity (11.1%), body trunk (5.6%)	Sprain (11.1%), fractures (5.6%)	3.1/1000 h
Surfing	Data not available	Data not available	Data not available
Table tennis	Data not available	Data not available	Data not available
Taekwondo [24]	Upper extremity (22.8%), lower extremity (9.1%)	Data not available	19.09/1000 AEs (competition)
Tennis [29]	Upper extremity (23.2%), lower extremity (51.4), body trunk (18.5%)	Muscle rupture/tear/spasm/cramp (32.9%), synovitis (20.4%), tendon tear/tendinopathy/bursitis (17.6%), ligament injury (8.3%), dislocation/subluxation/instability (6.0%), lesion of meniscus/articular cartilage (3.2%), fasciitis (2.3%), fracture (0.9%)	56.6/1000 h (95% CI: 49.5, 64.6) competition; 62.7/1000 h (95% CI: 54.8, 71.6) training
Triathlon [30]	Upper extremity/shoulder (up to 19%), lower extremity (36–85%), body trunk (up to 72%)	Muscle/tendon lesions (30–55%), tendinitis (13–25%), and ligament/joint injuries (6–29%)	17.4/1000 h (competition), 0.7–5.4/1000 h (training)
Volleyball (beach volleyball, volleyball [31])	Men: upper extremity (30.5%), lower extremity (46.3%), body trunk (12.2%) Women: upper extremity (19.2%), lower extremity (58.0%), body trunk (11.7%)	Men: sprains (22.0%), and strain (19.5%), inflammatory conditions (19.5%), fracture (6.1%), dislocation/subluxation (2.4%), entrapment/impingement (2.4%), sacroiliac dysfunction (1.2%), spasm (1.2%) Women: sprains (23.8%), strain (21.4%), inflammatory conditions (14.7%), fracture (2.8%), spasm (2.4%), entrapment/impingement (2.0%), patella femoral pain syndrome (1.8%), sacroiliac dysfunction (1.4%), dislocation/subluxation (1.0%)	Men: 4.69/1000 AEs (95% CI, 3.68–5.70) Women: 7.07/1000 AEs (95% CI, 6.45–7.68)
Weightlifting [32,33]	Upper extremity (39%), lower extremity (23%), body trunk (37%)	Data not available	2.4–3.3/1000 h (training)
Wrestling (Greco-Roman, freestyle) [34]	Upper extremity (23.1%), lower extremity (35.8%), body trunk (10.5%)	Knee internal derangements (18.9%), ankle ligament sprains (7.4%), shoulder strains (4.6%)	5.7/1000 AEs (95% CI 5.5–5.8) training; 26.4/1000 AEs (95% CI 25.4–27.3) competition

Notes: ^1^ IIRs are expressed in one of these 3 formats: (1) injuries/1000 h, (2) /1000 AEs, (3) 1000 player-hours. ^2^ Data are referred to as any kind of injury and not only to MSK-Is if not otherwise specified.

## Data Availability

Not applicable.
